# Exposure to wildfire smoke and risk of mortality and hospitalization among hemodialysis patients in the Western United States: Effect modification by extreme heat events

**DOI:** 10.1097/EE9.0000000000000541

**Published:** 2026-11-06

**Authors:** Hyeonjin Song, Nicole E. Sieck, Menglu Liang, Hao He, Evan Andrew Ellicott, Richard V. Remigio, Xin He, Quynh Nguyen, Xin-Zhong Liang, Jochen G. Raimann, Len A. Usvyat, Frank W. Maddux, Peter Kotanko, Amir Sapkota

**Affiliations:** a Department of Epidemiology and Biostatistics, University of Maryland School of Public Health, College Park, Maryland; b Department of Global, Environmental, and Occupational Health, University of Maryland School of Public Health, College Park, Maryland; c Department of Atmospheric and Oceanic Sciences, University of Maryland, College Park, Maryland; d Department of Geographical Sciences, University of Maryland, College Park, Maryland; e Department of Environmental, Agricultural, and Occupational Health, College of Public Health, University of Nebraska Medical Center, Nebraska Omaha; f Katz School of Science and Health, Yeshiva University, New York, New York; g Research Division, Renal Research Institute, New York, New York; h Fresenius Medical Care North America, Waltham, Massachusetts; i Icahn School of Medicine at Mount Sinai, New York, New York

**Keywords:** Extreme heat, Wildfire, End-stage kidney disease, Kidney failure

## Abstract

**Background:**

Climate change is increasing the extent of wildfires and extreme heat events (EHEs) in the Western United States. Our objective was to estimate the risk of all-cause mortality and hospitalization among end-stage kidney disease (ESKD) patients after exposure to wildfire smoke, EHEs, and the combined occurrence of wildfire smoke and EHE.

**Methods:**

We analyzed records of 55,223 adult ESKD patients who received hemodialysis treatment at Fresenius Kidney Care clinics in California, Oregon, and Washington during the warm months of 2010–2018. We quantified wildfire smoke plumes, wildfire-related particulate matter 2.5 (PM_2.5_), and EHE exposure for each hemodialysis facility. We employed a time-stratified case-crossover using conditional quasi-Poisson to estimate rate ratios (RRs) and 95% confidence intervals (CIs). A likelihood ratio test was used to assess potential interaction effects between wildfire smoke and EHEs, along with stratified analyses to estimate effect modification by EHEs.

**Results:**

The presence of a wildfire smoke plume was associated with a 19% increase in same-day (lag 0) risk of mortality (RR: 1.19; 95% CI: 1.11, 1.27), while a 10 μg/m^3^ increase in wildfire-related PM_2.5_ was associated with a 3% increase (RR: 1.03; 95% CI: 1.02, 1.06) in mortality risk. A 10 μg/m^3^ increase in wildfire-related PM_2.5_ was associated with a 1% increase in the risk of hospitalization (RR: 1.01; 95% CI: 1.00, 1.03) at lag 0. We observed considerably higher risk of mortality when patients were simultaneously exposed to wildfire smoke plume and EHE (RR: 1.92; 95% CI: 1.71, 2.17) compared with wildfire smoke plume alone (RR: 1.02; 95% CI: 0.95, 1.10). We did not observe significant effect modification by EHE for all-cause hospitalization.

**Conclusions:**

These findings suggest that patients with ESKD are at a higher risk of mortality after exposure to wildfire smoke, particularly in the presence of EHEs.

**What this study adds:**

Exposure to both extreme heat and wildfire smoke is projected to become more common in a changing climate. While prior studies have documented their risks separately, there is a paucity of data regarding their joint impact among vulnerable populations such as ESKD patients. In this study, we observed that ESKD patients were at a greater risk of mortality and hospitalization when exposed to wildfire smoke. The risk of mortality was considerably higher when simultaneously exposed to both wildfires and extreme heat.

## Introduction

The Fifth National Climate Assessment concludes that the frequency and intensity of extreme heat events (EHEs) are increasing in the United States,^1^ a trend that will likely continue in response to ongoing climate change.^2^ Heat exposure represents the leading cause of weather-related deaths in the United States, with an average of 702 deaths annually between 2004 and 2018.^3^ Heatwaves, along with droughts, increase the risk of wildfires by contributing to the drying of vegetation, which subsequently promotes the rapid spread of wildfires.^4,5^ Since the 1980s, the Western United States has experienced continuous warming accompanied by an increased frequency of wildfires and a lengthening of the fire season, with many global climate models suggesting this trend will continue.^6,7^ The National Interagency Coordination Center reported that, on average over 2014–2024, there were 62,307 wildfires that burned approximately 7 million acres annually in the United States, with the Western United States (Washington, Oregon, and California) accounting for one-third of the nation’s total acres burned.^8^

Smoke plumes from wildfires can travel over long distances, impacting communities that are geographically distant from the fire sources.^9–11^ For example, air pollutants from 2023 wildfires in Southern Canada contributed to regions in the Eastern United States exceeding the National Ambient Air Quality Standard for fine particulate matter (aerodynamic diameter <2.5 microns; PM_2.5_).^12,13^ Wildfire smoke contains high concentrations of hazardous air pollutants, including PM_2.5_, carbon monoxide, nitrogen dioxide, volatile organic compounds, and polycyclic aromatic hydrocarbons.^5,14^ In comparison to urban ambient PM, wildfire-related PM is predominantly carbonaceous and contains higher levels of free radicals, increasing its potential to induce inflammatory and oxidative responses.^15,16^ Toxicological studies have demonstrated that PM from wildfires is more toxic than PM from other sources.^17,18^ Therefore, it is important to differentiate wildfire-related PM from background PM when assessing its health impacts.^19^

Communities across the United States are increasingly facing threats from climate change–related compound hazards where two or more hazards occur simultaneously or in close succession.^1^ A systematic review of 56 studies involving compound hazards concluded that there is sufficient evidence supporting the synergistic effects of heat and air pollution exposure.^20^ The authors underscored the need for research investigating the potential impact of compound hazards, given that individuals are often exposed to multiple environmental risk factors simultaneously. However, there is a paucity of data regarding whether such compound hazards disproportionately impact individuals living with end-stage kidney disease (ESKD).^21^ Extreme heat can disproportionately impact ESKD patients because their liquid intake is strictly regimented; therefore, they cannot cope with the higher temperature by increasing their liquid intake like healthy individuals.^22,23^ Previous research provides evidence for increased risk of both hospitalization and mortality after exposure to extreme heat.^24,25^ Similarly, exposure to wildfire smoke has been shown to increase the risk of mortality as well as respiratory and cardiovascular complications among hemodialysis patients.^11,26^

With projected increases in wildfire activity and EHEs in the Western United States, there is a need to investigate how such compound hazards impact mortality and hospitalization risk among ESKD patients. This study aims to bridge these knowledge gaps by examining (1) how exposure to wildfire-related air pollution (wildfire smoke plumes and wildfire-related PM_2.5_) and EHEs impacted the risks of mortality and hospitalization among hemodialysis patients, and (2) the potential effect modification by EHEs on the association between wildfire-related air pollution exposure and the risks of mortality and hospitalization. We hypothesize that exposure to wildfire-related air pollution and EHEs increases the risks of mortality and hospitalization among ESKD patients, and that compound hazards consisting of both wildfire and EHE are more detrimental to ESKD patients.

## Methods

### Study Population and Health Outcomes

We obtained deidentified electronic health records of in-center hemodialysis patients aged 18 or older who underwent hemodialysis treatment between 2010 and 2018 at Fresenius Kidney Care (FKC) clinics located in the Western United States (Washington, Oregon, and California). Because one of the main exposure variables was EHEs, we restricted the study to warmer months spanning from May to September, beginning in June 2010. The relevant health records included information on each patient’s age, sex, race/ethnicity, identification code of the treatment clinic visited, and the dates of all-cause hospitalizations and deaths. All-cause hospitalizations were included in this study without restrictions. This study was approved by the University of Maryland Institutional Review Board (Exempt Category #4). We followed the STrengthening the Reporting of OBservational studies in Epidemiology guidelines for cohort studies.^27^

### Exposure

#### Presence of wildfire smoke plume and wildfire-related PM_2.5_ concentrations

We quantified wildfire-related air pollution exposure at each hemodialysis clinic using two exposure metrics: (1) wildfire smoke plume and (2) wildfire-related PM_2.5_ concentration.^11^ To assess for the presence of a wildfire smoke plume on a given day, we overlaid the clinic’s geocoordinates with the Hazard Mapping System (HMS) from the National Oceanic and Atmospheric Administration, which is a satellite image display system that provides wildfire information for North America (Figure 1).^28,29^ HMS classifies the density of smoke within its polygons as light, medium, and heavy based on the opacity of satellite images. A recent remote sensing study suggested that restricting the HMS polygons to medium and heavy smoke categories more closely represents surface smoke.^30^ Hence, we established a binary exposure indicator for “wildfire smoke plume” for each clinic on a daily basis, assigning it a value of 1 when the HMS polygon’s density was classified as “medium” or “heavy” smoke, and 0, if otherwise.

**Figure 1.**
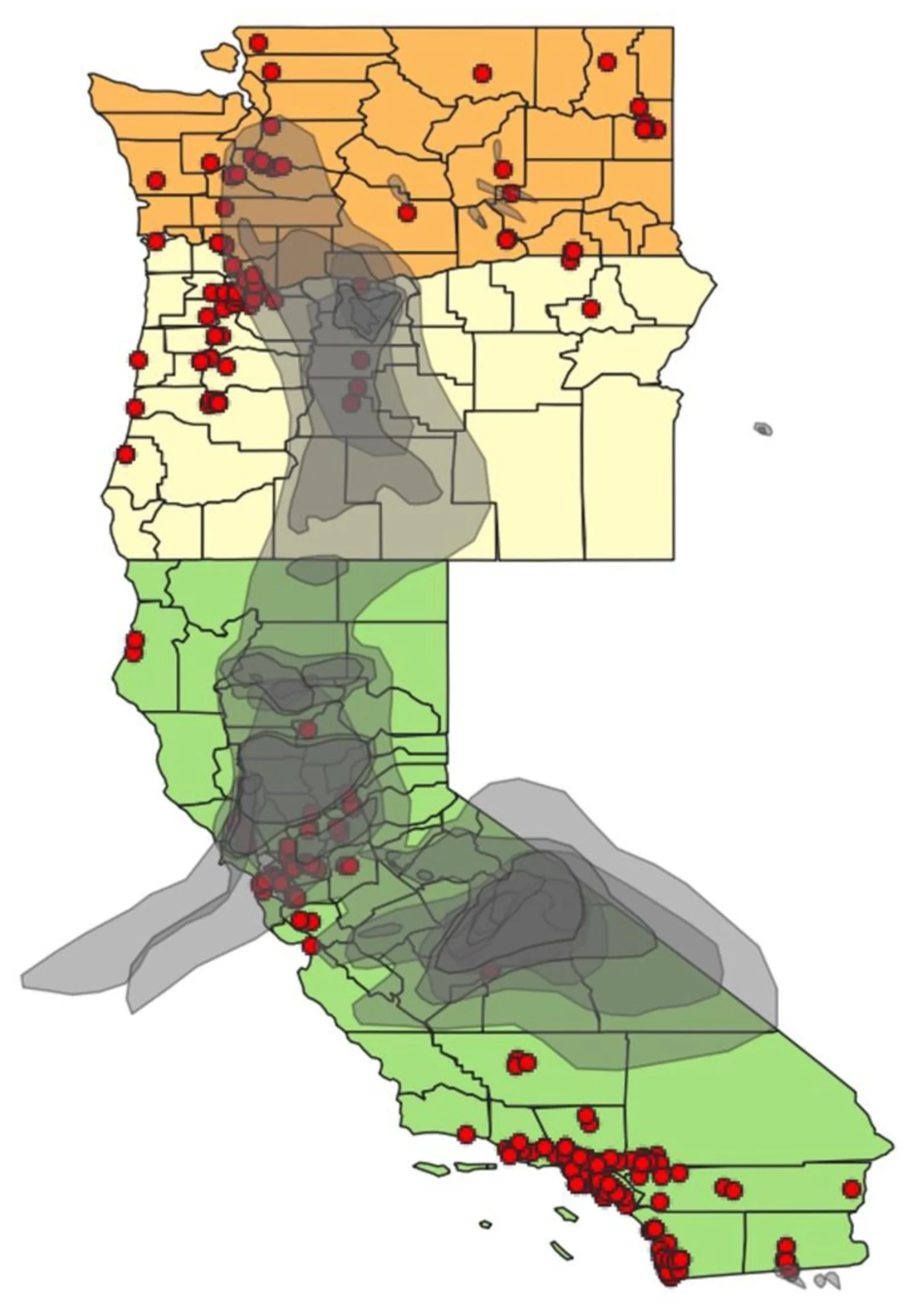
Hemodialysis clinics in the Western United States (Washington, Oregon, and California) and the Hazard Mapping System (HMS) image depicting the presence of wildfire smoke plume on June 24, 2018. Red circles indicate the locations of the included hemodialysis clinics. The gray shaded areas represent the HMS smoke plume polygons on June 24, 2018. The states are color-coded as follows: orange for Washington, yellow for Oregon, and green for California.

To differentiate wildfire-related PM_2.5_ from the overall PM_2.5_ originating from other sources, we utilized a multistep approach employing daily ambient PM_2.5_ concentration data from the U.S. Environmental Protection Agency Air Quality System (AQS), after a previously established method.^31–33^ We matched each hemodialysis clinic with all PM_2.5_ monitors within a 20-km radius, then averaged PM_2.5_ concentrations from different AQS monitors within the 20-km radius for each clinic-day. A 20-km radius was selected based on prior epidemiologic literature as a compromise between accuracy in exposure assessment and inclusion of a majority of the hemodialysis clinics.^31^ If a given clinic was not under a wildfire smoke plume polygon, the estimated wildfire-related PM_2.5_ for that clinic was assigned a value of 0 μg/m^3^, while non-wildfire-related PM_2.5_ was assigned the recorded ambient PM_2.5_ concentrations. When a clinic fell under a wildfire smoke plume, the estimated wildfire-related PM_2.5_ concentration for that specific day was calculated by subtracting the seasonal nonsmoke background PM_2.5_ concentration from the ambient PM_2.5_ concentration for that given day. Seasonal nonsmoke background PM_2.5_ concentrations were computed as the median of the ambient PM_2.5_ concentrations of sites without wildfire smoke plumes for each season (Spring: March–May, Summer: June–August, and Fall: September–November) within the same year. In instances where the ambient PM_2.5_ concentration was lower than the seasonal non-smoke background PM_2.5_, a value of 0 μg/m^3^ was assigned for the wildfire-related PM_2.5_. Non-wildfire-related PM_2.5_ was computed by subtracting the wildfire-related PM_2.5_ from the total ambient PM_2.5_ concentration. Of 190 FKC clinics in the Western United States, 15 clinics were excluded from the analyses because they were without an air pollution monitoring station within a 20-km radius. An additional 14 clinics were excluded due to having a high percentage of missing concentration data for PM_2.5_ (>30%).

### Extreme heat event

To identify EHEs for each clinic, we utilized the daily maximum Universal Thermal Climate Index (UTCI_max_). The UTCI is a bioclimatic index assessing human thermal comfort by accounting for the combined effects of air temperature, wind speed, humidity, and mean radiant temperature.^34,35^ In this study, EHE exposure was assigned to each clinic using previously described methods.^21,36^ Briefly, 30-year baseline daily UTCI_max_ data from 1980 to 2009 were used to determine a 95th percentile threshold for each calendar day and clinic. This was accomplished by employing a 31-day moving window centered around the date of interest. The daily UTCI_max_ for each clinic and calendar day during the study period was compared with each clinic’s respective 95th percentile thresholds and assigned a value of 1 (EHE) if it exceeded the clinic and calendar day-specific thresholds. Use of such a metric accounts for differences in climate between included clinics, which range from warm-summer Mediterranean, temperate oceanic, cold semi-arid, to hot desert climates after the Köppen–Geiger classifications.^37^

### Statistical Analysis

We conducted a time-stratified case-crossover analysis to examine the association of wildfire exposure and EHEs with all-cause mortality and all-cause hospitalization risk.^38,39^ The exposure status of the individual during the case period is compared with the exposure status of the same individuals during the control periods, which controls for all time-invariant individual-level confounders including sex, age, and race/ethnicity.^40,41^ The case-crossover design is particularly suitable for investigating transient effects of intermittent exposures on acute outcomes.^40^

We used conditional quasi-Poisson regression, which models the rate of events during each time period as a function of the exposure variable and permits adjustment of overdispersion, autocorrelation, and varying rate denominators, an alternative to conditional logistic regression.^42^ The number of daily deaths and hospitalizations were used as the outcome variables, while wildfire smoke plume, wildfire-related PM_2.5_ concentrations, and EHEs served as the three main exposure variables. For the analyses involving exposure to wildfire smoke plume and wildfire-related PM_2.5_, we adjusted for non-wildfire-related PM_2.5_ as a potential confounder. To adjust for varying populations, we included an offset variable equivalent to the natural logarithm of the monthly number of hemodialysis patients for each clinic. Measures of associations were reported as rate ratios (RRs) with 95% confidence intervals (95% CIs). The RRs for wildfire-related PM_2.5_ were expressed as 10-μg/m^3^ increases. Furthermore, we employed the unconstrained distributed lag model framework to assess lag effects for up to 1 day after the exposure. In this case, “lag 0” represents the risk associated with the same day as the exposure, while “lag 1” pertains to the risk 1 day after exposure.^43^ We selected this lag window based on our earlier research in this patient population, which suggested the largest risk of all-cause mortality and hospitalization outcomes within 1 day post-exposure in patients with ESKD after wildfire smoke exposure.^11^ Our statistical models took the following form:


$$\begin{array}{c} \begin{array}{cc} & Log\left(E\left(Y_{t,s=clinic\times month\times year\times DOW}\right)\right)\\ & =\alpha_{s}+\beta_{1}\left(wf\right)+\beta_{2}\left(EHE\right)+\beta_{3}\left(nwf\right)+\beta_{4}\left(wf\times EHE\right) \end{array} \end{array}$$

where *Y*_*t,s*_ is the outcome (mortality or hospitalization) on day *t,* assumed to follow a quasi-Poisson distribution conditioned on the sum of events in each stratum, *s*, defined by clinic, month, year, and day of week (DOW). Separate models were run for lag 0 and lag 1; thus, exposure effects were assessed for their occurrence on the same lag day. We examined the potential effect modification by EHEs on the association of exposure to wildfire smoke plume and wildfire-related PM_2.5_ with mortality and hospitalization. We compared the models with and without an interaction term using likelihood ratio tests. Subsequently, we stratified the analysis for wildfire and adverse outcome by EHE status (presence/absence).

We performed a sensitivity analysis using all smoke plume densities, assigning a value of 1 for the presence of wildfire smoke plume when the HMS polygon’s density was classified as “light,” “medium” or “heavy,” and 0, if otherwise. Wildfire-related PM_2.5_ concentrations were then estimated for days added to the analyses with “light” smoke density. We computed descriptive statistics to compare the exposure profiles between the clinics excluded due to missing PM_2.5_ monitor data, with the included clinics and ran analyses using non-wildfire-related PM_2.5_. In addition to the multiplicative interaction test for the main analyses, we assessed additive interaction measures and reported the relative excess risk due to interaction (RERI) and the corresponding attributable proportion. For our analyses, we utilized R statistical software version 3.6.1 with the *dlnm*, *dplyr*, and *gnm* packages.^43–46^ All statistical tests were two-tailed and based on a significance level of 0.05.

## Results

This study included 55,223 patients across 161 hemodialysis clinics within California, Oregon, and Washington (Table 1). There were 5379 all-cause deaths and 61,627 all-cause hospitalizations during the study period. Approximately 50.6% of the study population were 18–64 years old at hemodialysis initiation, 58.4% were male, and 42.0% were non-Hispanic White.

**Table 1.** Characteristics of study population

Characteristics	
Clinics, n	161
Patients, n	55,223
All-cause mortality, n	5,379
All-cause hospitalization, n	61,627
Age at initial treatment, n (%)	
18–64	27,958 (50.6)
65–74	13,209 (23.9)
≥75	11,588 (21.0)
Not reported	2,468 (4.5)
Sex, n (%)	
Female	22,998 (41.6)
Male	32,225 (58.4)
Race/ethnicity, n (%)	
Hispanic	10,242 (18.5)
Non-Hispanic Black	5,230 (9.5)
Non-Hispanic White	23,199 (42.0)
Asian American	3,050 (5.5)
Other	965 (1.7)
Not reported	12,537 (22.7)

Table 2 provides a summary of daily total PM_2.5_, wildfire-related PM_2.5_ concentrations, non-wildfire-related PM_2.5_ concentrations, and daily UTCI_max_ for each clinic. The daily total PM_2.5_ concentrations for each hemodialysis clinic ranged from 0 to 485.8 μg/m^3^, with a mean of 10.2 μg/m^3^. The daily wildfire-related PM_2.5_ concentrations ranged from 0 to 480.8 μg/m^3^, with a mean of 13.7 μg/m^3^. The daily non-wildfire-related PM_2.5_ concentrations varied between 0 and 130.6 μg/m^3^, with a mean of 9.8 μg/m^3^. The daily UTCI_max_ ranged from −5.2 to 56.1 °C, with a mean of 30.8 °C. The mean number of days with wildfire smoke plume per month per clinic was 1 day. Meanwhile, each clinic had a mean of two days of EHE exposure per month. Additional descriptive statistics regarding the exposures are presented in the supplemental materials (eTable 1, https://links.lww.com/EE/A493).

**Table 2.** Daily measures of PM_2.5_ concentrations and daily measures of UTCI_max_ for each clinic in the Western United States (June 2010–September 2018) during the warm season (May–September)

		Mean (SD)	Min	Med	Max
Daily PM_2.5_ (μg/m^3^)	Total PM_2.5_	10.2 (7.7)	0	9.3	485.8
	Wildfire-related PM_2.5_^a^	13.7 (24.8)	0	5	480.8
	Non-wildfire-related PM_2.5_^b^	9.8 (5.7)	0	9.2	130.6
Daily UTCI_max_ (°C)		30.8 (6.5)	-5.2	30.7	56.1
Number of wildfire smoke days per month per clinic		1 (3)	0	0	28
Number of EHE days per month per clinic		2 (2)	0	1	14

a During the days with a wildfire smoke plume (6,289 clinic-days).

b During the days without a wildfire smoke plume (149,676 clinic-days).

Table 3 shows the results stratified by the presence of EHEs and wildfire smoke plumes. A total of 1,538 (1.0%) clinic-days experienced joint occurrences of wildfire smoke plumes and EHEs, while 90.0% of clinic-days had neither a wildfire smoke plume nor an EHE. The average total PM_2.5_ concentration was highest when both wildfire smoke plumes and EHEs were present (25.8 μg/m^3^). Likewise, the mean wildfire PM_2.5_ concentration was higher during EHE days compared with non-EHE days (19.0 μg/m^3^ vs. 12.0 μg/m^3^). Mean UTCI_max_ was higher during wildfire smoke plume days compared with non-wildfire smoke plume days (39.2 °C vs. 38.6 °C during EHE days and 33.2 °C vs. 30.1 °C during non-EHE days). The mortality rate was highest when both a wildfire smoke plume and EHE were present, while the hospitalization rate was slightly lower when both a wildfire smoke plume and EHEs were present.

**Table 3.** Summary of daily mean PM_2.5_ concentrations, UTCI_max_, and health outcomes stratified by the presence of EHEs and wildfire smoke plume

	N clinic-days	Total PM_2.5_ concentration	Wildfire PM_2.5_ concentration	UTCI_max_	Monthly mortality rate	Monthly hospitalization rate
WF=0, EHE=0	140,394	9.6	-	30.1	3.4	56.4
WF=0, EHE=1	9,282	11.6	-	38.6	3.4	56.0
WF=1, EHE=0	4,751	18.1	12.0	33.2	4.1	56.9
WF=1, EHE=1	1,538	25.8	19.0	39.2	5.1	53.3

WF=0, days without a wildfire smoke plume; WF=1, days with a wildfire smoke plume; EHE=0, days without an EHE; EHE=1, days with an EHE; Mortality rate: 10,000 × (Daily number of deaths/monthly number of hemodialysis patients who visited the clinic); Hospitalization rate: 10,000 × (daily number of hospitalizations/monthly number of hemodialysis patients who visited the clinic)

The presence of wildfire smoke was associated with a 19% (lag 0) increase in mortality risk (RR_Lag0_: 1.19; 95% CI: 1.11, 1.27) and a 4% increase in hospitalization risk (lag 1) that was borderline significant (RR_Lag1_: 1.04; 95% CI: 0.99, 1.09) after adjusting for non-wildfire-related PM_2.5_ (Table 4). A 10-μg/m^3^ increase in wildfire-related PM_2.5_ was associated with a 3% increase in risk of mortality (RR_Lag0_: 1.03; 95% CI: 1.02, 1.06) and a 1% increase in hospitalization risk (RR_Lag0_: 1.01; 95% CI: 1.00, 1.03). Exposure to EHEs was associated with a 5% higher risk (lag1) of mortality (RR_Lag1_: 1.05; 95% CI: 1.00, 1.10) and a 3% higher risk of hospitalization on the same day as exposure (RR_Lag0_: 1.03; 95% CI: 1.00, 1.06). We performed sensitivity analysis by including the “light” smoke plumes in the exposure metric (eTable 2, https://links.lww.com/EE/A493), which decreased the effect estimates.

**Table 4.** Rate ratios and 95% confidence intervals for risk of mortality and hospitalization associated with exposure to wildfire smoke plume, wildfire-related PM_2.5_, and EHEs

	Lag	Mortality	Hospitalization
Presence of wildfire smoke plume^a^	Lag 0	1.19 (1.11, 1.27)	1.01 (0.96, 1.06)
	Lag 1	1.03 (0.96, 1.10)	1.04 (0.99, 1.09)
Wildfire-related PM_2.5_ 10 μg/m^3^ increase^a^	Lag 0	1.03 (1.02, 1.06)	1.01 (1.00, 1.03)
	Lag 1	1.02 (1.00, 1.04)	1.01 (0.99, 1.03)
EHEs	Lag 0	1.04 (0.99, 1.08)	1.03 (1.00, 1.06)
	Lag 1	1.05 (1.00, 1.10)	0.99 (0.96, 1.02)

a Adjusted for non-wildfire-related PM_2.5._

Model comparison test results examining the inclusion of interaction terms and EHE-stratified analyses are shown in Table 5. For mortality, we observed a significant interaction between EHEs and wildfire smoke plumes at both lag 0 and 1. A higher RR of smoke plume-related mortality was observed during EHE days (RR_Lag0_: 1.92; 95% CI: 1.71, 2.17) relative to non-EHE days (RR_Lag0_: 1.02; 95% CI: 0.95, 1.10). Likewise, the RR for mortality associated with a 10-μg/m^3^ increase in wildfire-related PM_2.5_ was more pronounced during EHE days (RR_Lag0_: 1.08; 95% CI: 1.05, 1.11) compared with non-EHE days (RR_Lag0_: 1.00; 95% CI: 0.98, 1.03). We did not observe significant interactions for all-cause hospitalization. Our sensitivity analysis, including light-density smoke plumes, yielded lower effect estimates (eTable 3, https://links.lww.com/EE/A493). In eTable 4, https://links.lww.com/EE/A493, we report total PM_2.5_ concentrations by plume density. The associations for all-cause mortality and hospitalization associated with non-wildfire-related PM_2.5_ are presented in eTable 5, https://links.lww.com/EE/A493. Exposure profiles for the included and excluded clinics are reported in eTable 6, https://links.lww.com/EE/A493. Our analysis to assess for an additive interaction suggested that nearly half of the mortality risk on days with both exposures was attributable to their interaction rather than to either exposure alone (eTable 7, https://links.lww.com/EE/A493).

**Table 5.** Risks of mortality and hospitalization associated with exposure to wildfire (wildfire smoke plume and wildfire-related PM_2.5_), stratified by EHE

Outcome	Exposure	Lag	RR (95% CI) adjusted for EHE	Interaction with EHE		EHE stratification	
				*F*-test, df	*P* value	EHE days	Non-EHE days
Mortality	Wildfire smoke plume	Lag 0	1.18 (1.10, 1.26)	*F*(83.7), 1	<0.01	1.92 (1.71, 2.17)	1.02 (0.95, 1.10)
		Lag 1	1.02 (0.95, 1.09)	*F*(69.8), 1	<0.01	1.55 (1.38, 1.74)	0.88 (0.81, 0.95)
	Wildfire-related PM_2.5_	Lag 0	1.03 (1.01, 1.05)	*F*(14.3), 1	<0.01	1.08 (1.05, 1.11)	1.00 (0.98, 1.03)
		Lag 1	1.02 (1.00, 1.04)	*F*(31.3), 1	<0.01	1.09 (1.06, 1.12)	0.98 (0.95, 1.00)
Hospitalization	Wildfire smoke plume	Lag 0	1.00 (0.96, 1.05)	*F*(0.27), 1	0.60	0.98 (0.90, 1.08)	1.01 (0.96, 1.06)
		Lag 1	1.04 (1.00, 1.09)	*F*(3.28), 1	0.07	1.12 (1.02, 1.22)	1.02 (0.97, 1.08)
	Wildfire-related PM_2.5_	Lag 0	1.01 (0.99, 1.03)	*F*(1.22), 1	0.27	1.00 (0.98, 1.03)	1.02 (1.00 1.04)
		Lag 1	1.01 (0.99, 1.03)	*F*(2.13), 1	0.14	1.00 (0.97, 1.02)	1.02 (1.00, 1.04)

All models adjusted for non-wildfire-related PM_2.5_.

## Discussion

In this large study of ESKD patients receiving in-center hemodialysis treatments, we observed a significant interactive effect of EHEs and wildfire-related air pollution exposure on the risk of all-cause mortality. Furthermore, we observed that wildfire smoke plume-related risk of mortality was considerably higher when the smoke plume and EHE occurred simultaneously (RR_Lag0_: 1.92; 95% CI: 1.71, 2.17) as opposed to the smoke plume alone (RR _Lag0_: 1.02; 95% CI: 0.95, 1.10). A similar pattern, albeit with lower effect estimates, was observed using the wildfire-related PM_2.5_ metric. The findings of smoke exposure on non-EHE days warrant further investigation, particularly at lag 1, where we observed a reduction in risk associated with wildfire smoke plume exposure (RR_Lag1_: 0.88; 95% CI: 0.81, 0.95). We did not observe a significant difference in the risk of hospitalization between EHE and non-EHE days, nor was the presence of a wildfire smoke plume significantly associated with the risk of hospitalization. The unexpected null findings for the risk of hospitalization may be due to excess mortality associated with wildfire smoke exposure, though this explanation requires additional analysis. Alternatively, disruptions in healthcare access due to the occurrence of wildfires or extreme heat, an insufficient lag window, or exposure misclassification may have contributed to the null findings. Likewise, the inclusion of all-cause hospitalization may dilute an effect to specific hospitalization causes associated with exposure to wildfire smoke and extreme heat.

Our novel findings regarding the joint impact of wildfire exposure and EHEs build upon prior studies that have looked at these risk factors separately. Remigio et al.^24^ investigated the impact of EHEs on ESKD patients receiving in-center hemodialysis treatments at FKC clinics in Boston, New York, and Philadelphia from 2001 to 2012, reporting 27% and 31% higher risk of hospitalization and all-cause mortality, respectively. In subsequent expanded studies that included data from 28 northeastern US counties spanning from 2001 to 2016, the authors reported a 5% increased risk of all-cause mortality for every 10-μg/m^3^ increase in PM_2.5_ (cumulative RR for Lag_0-3_: 1.05; 95% CI: 1.00,1.10) among ESKD patients.^21^ In a study of the impact of the 2023 Canadian wildfires on patients with ESKD who received treatment at FKC clinics located in the Eastern and Midwestern United States, researchers observed significant increases in the risk of all-cause mortality, all-cause hospitalization, and respiratory hospitalizations.^11^ Although the estimates of the risk of all-cause mortality were similar to those reported in the present study, the risk of all-cause hospitalization was higher (RR_Lag0_: 1.33; 95% CI: 1.10, 1.62), which may be due in part to the intensity of the wildfire smoke after this event.^11^ In another study using health records of hemodialysis patients in 253 counties across the United States, obtained from the USRDS for the years 2008–2012, Xi et al.^26^ reported that a 10-μg/m^3^ increase in wildfire-related PM_2.5_ was associated with a 4% increase in all-cause mortality on the same day (RR_Lag0_: 1.04; 95% CI: 1.01, 1.07), similar to what we observed in this study.

To our knowledge, this is the first study to investigate the combined effect of wildfire smoke plume, wildfire-related PM_2.5_, and EHEs among hemodialysis patients. Previous research has mainly focused on examining the interaction between extreme heat and air pollution, not wildfire smoke plume specifically.^21,47,48^ Additionally, our study population was treated at clinics operated by FKC, a prominent private provider of dialysis services in North America, treating approximately 38% of all hemodialysis patients.^49^ Despite this large coverage, the patient population may not be a representative sample of all ESKD patients in the United States.

One limitation of this study is the potential for exposure misclassification regarding wildfire smoke plumes. HMS relies on satellite observation and detects smoke plumes from the ground to high altitudes; thus, it may not accurately represent air quality near the ground. However, a remote sensing study suggested that limiting the HMS smoke polygons to medium and heavy smoke closely represents surface smoke.^30^ The results of our study exhibited more pronounced mortality risk when restricted to medium and heavy smoke, and we observed an increasing trend in PM_2.5_ concentrations with more dense smoke plumes. We may have underestimated wildfire-related PM_2.5_ exposure by assigning wildfire-related PM_2.5_ exposure as “0” when clinics were not under the presence of a medium or heavy plume. There is emerging evidence that wildfire-related PM_2.5_ may be underestimated, particularly far downwind.^50^ Wildfire smoke contains a complex mixture of pollutants (e.g., carbon monoxide, nitrogen dioxide, polycyclic aromatic hydrocarbons, heavy metals),^16^ so we cannot rule out the impact of exposure to these other pollutants in our estimations of the association of wildfire-related PM_2.5_. Finally, the spatial resolution for exposure assessment was at the clinic level, as we did not have access to residential addresses. Such an approach does not capture the true heterogeneity in exposure that exists between patients receiving treatment in the same clinic, particularly if the catchment area of the clinic is large enough for smoke plume heterogeneity to exist, i.e., if patients seen at a given clinic are commuting in for treatment from potentially long distances. Regardless, such measurement errors, if present, were likely to be nondifferential, as exposure measurement errors would be independent of outcome measurement, which would attenuate the risk estimates.^51^

A growing body of literature provides evidence on the public health threats posed by ongoing climate change.^52^ While this literature has mostly focused on how single hazards exacerbated by climate change impact health, there remains a notable gap in research when it comes to understanding how compound hazards impact health. Given that such compound hazards are projected to increase in frequency and intensity into the foreseeable future,^1,2^ there is an urgent need to develop early warning systems that can inform clinic-level protocols designed to safeguard hemodialysis patients. Effective early warning systems should take into consideration current weather and wildfire conditions combined with seasonal forecasting, along with individual- and area-level characteristics to provide a meaningful outlook to inform clinic-level decision-making. These systems should also facilitate communication regarding risks and prevention strategies while continuously evaluating and refining their effectiveness.^52,53^ Such effort may contribute to reducing treatment costs for ESKD patients, which reached approximately $55.3 billion in the United States in 2023.^54^

## ACKNOWLEDGMENTS

We thank Sheetal Chaudhuri for performing data processing and extraction from the Fresenius Kidney Care data sources.

## Supplementary Material


