# Supplementary material for: Exposure to wildfire smoke and risk of mortality and hospitalization among hemodialysis patients in the Western United States: Effect modification by extreme heat events
Source: Environ Epidemiol. 2026 Nov 6;10(6):e541. doi: 10.1097/EE9.0000000000000541 (PMC13747479; doi:10.1097/EE9.0000000000000541)
Supplement: Supplementary file 1 [file ee9-10-e541-s001.pdf]

# Supplemental Materials

## Table of Contents


***eTable 1*** ........................................................................................................................ *2*

***eTable 2*** ........................................................................................................................ *3*

***eTable 3*** ........................................................................................................................ *4*

***eTable 4*** ........................................................................................................................ *5*

***eTable 5*** ........................................................................................................................ *6*

***eTable 6*** ........................................................................................................................ *7*

***eTable 7*** ........................................................................................................................ *8*

## eTable 1. Descriptive statistics across joint exposure categories.

Wildfire smoke plume and wildfire-related $PM_{2.5}$ estimated using medium and heavy densities of smoke plume.

| Category | Clinic-days | % | Clinics | Deaths | Hosp. | WF $PM_{2.5}$ mean (SD) | $UTCI_{max}$ mean (SD) |
|---|---|---|---|---|---|---|---|
| **Neither** | 140,394 | 90.02 | 161 | 4,802 | 55,286 | 0.00 (0.00) | 30.1 (6.2) |
| **Smoke plume only** | 4,751 | 3.05 | 161 | 178 | 1,711 | 12.0 (23.6) | 33.2 (5.5) |
| **EHE only** | 9,282 | 5.95 | 161 | 305 | 3,814 | 0.00 (0.00) | 38.6 (5.2) |
| **Both** | 1,538 | 0.99 | 160 | 69 | 548 | 19.0 (27.6) | 39.2 (4.0) |

**eTable 2. Rate ratios and 95% confidence intervals for risk of mortality and hospitalization associated with exposure to wildfire smoke plume, wildfire-related $PM_{2.5}$, and EHEs.** Wildfire smoke plume and wildfire-related $PM_{2.5}$ estimated using all densities of smoke plume including light, medium, and heavy.

| | Lag | Mortality | Hospitalization |
|---|---|---|---|
| Presence of wildfire smoke plume[a] | $Lag_0$ | 1.02 (0.98, 1.06) | 1.02 (0.99, 1.04) |
| | $Lag_1$ | 1.02 (0.98, 1.06) | 1.04 (1.01, 1.06) |
| Wildfire-related $PM_{2.5}$ 10 $\mu g/m^3$ increase[a] | $Lag_0$ | 1.02 (1.00, 1.04) | 1.01 (1.00, 1.03) |
| | $Lag_1$ | 1.01 (0.99, 1.03) | 1.01 (1.00, 1.03) |
| EHEs | $Lag_0$ | 1.04 (0.99, 1.08) | 1.03 (1.00, 1.06) |
| | $Lag_1$ | 1.05 (1.00, 1.10) | 0.99 (0.96, 1.02) |

[a]Adjusted for non-wildfire-related $PM_{2.5}$

**eTable 3. Risks of mortality and hospitalization associated with exposure to wildfire (wildfire smoke plume and wildfire-related $PM_{2.5}$), stratified by extreme heat event (EHE).** Wildfire smoke plume and wildfire-related $PM_{2.5}$ estimated using all types of smoke plume including light, medium, and heavy. All models adjusted for non-wildfire-related $PM_{2.5}$.

| Outcome | Exposure | Lag | RR (95% CI) adjusted for EHE | Interaction with EHE | | EHE Stratification | |
|---|---|---|---|---|---|---|---|
| | | | | F-test, df | *p*-value | EHE Days | Non-EHE Days |
| Mortality | Wildfire smoke plume | Lag 0 | 1.02 (0.98, 1.06) | F(39.5), 1 | <0.01 | 1.34 (1.22 1.47) | 0.98 (0.94, 1.02) |
| | | Lag 1 | 1.02 (0.98, 1.06) | F(62.0), 1 | <0.01 | 1.43 (1.30, 1.56) | 0.96 (0.92, 1.00) |
| | Wildfire-related $PM_{2.5}$ | Lag 0 | 1.02 (1.00, 1.04) | F(18.3), 1 | <0.01 | 1.07 (1.04, 1.10) | 0.99 (0.96, 1.01) |
| | | Lag 1 | 1.01 (0.99, 1.03) | F(48.7), 1 | <0.01 | 1.08 (1.06, 1.12) | 0.95 (0.93, 0.98) |
| Hospitaliz-ation | Wildfire smoke plume | Lag 0 | 1.01 (0.99, 1.04) | F(0.04), 1 | 0.84 | 1.02 (0.96, 1.09) | 1.01 (0.99, 1.04) |
| | | Lag 1 | 1.04 (1.01, 1.07) | F(1.26), 1 | 0.26 | 1.07 (1.01, 1.15) | 1.03 (1.00, 1.06) |
| | Wildfire-related $PM_{2.5}$ | Lag 0 | 1.01 (1.00, 1.03) | F(1.25), 1 | 0.26 | 1.00 (0.98, 1.03) | 1.02 (1.00, 1.04) |
| | | Lag 1 | 1.02 (1.00, 1.03) | F(2.95), 1 | 0.09 | 1.00 (0.98, 1.02) | 1.03 (1.01, 1.05) |

## eTable 4. $PM_{2.5}$ by smoke plume density.

We calculated the total PM2.5 mean concentrations when no plume was present, and for each of the three Hazard Mapping System (HMS) categories: light, medium, and heavy. We further report the median and associated interquartile range (IQR) and the percentage of days exceeding 35 µg/m³, the regulatory threshold per the National Ambient Air Quality Standards (NAAQS) set the by US Environmental Protection Agency (EPA).

| Plume density | Clinic-days | Total $PM_{2.5}$ mean | Median (IQR) | % days > 35 µg/m³ |
|---|---|---|---|---|
| **No plume** | 134,504 | 9.63 | 9.05 (7.70) | 0.17 |
| **Light** | 15,172 | 10.90 | 10.15 (7.90) | 0.65 |
| **Medium** | 3,788 | 14.44 | 11.20 (9.65) | 6.62 |
| **Heavy** | 2,501 | 28.46 | 17.05 (25.92) | 26.41 |

**eTable 5. Association of outcomes with non-wildfire-related $PM_{2.5}$.**

Values represent the rate ratios with those in parentheses corresponding 95% confidence intervals.

| Outcome | Lag 0 | Lag 1 | Lag 2 | Lag 3 | Cumulative 0–3 |
|---|---|---|---|---|---|
| **All-cause mortality** | 1.02 (0.99, 1.06) | 1.01 (0.97, 1.04) | 1.02 (0.98, 1.05) | 1.00 (0.97, 1.03) | 1.05 (1.00, 1.09) |
| **All-cause hospitalization** | 0.99 (0.97, 1.01) | 0.99 (0.97, 1.02) | 1.03 (1.01, 1.06) | 0.97 (0.95, 1.00) | 0.99 (0.96, 1.02) |

## eTable 6. Descriptive statistics for included and excluded facilities.

Wildfire smoke plume and wildfire-related $PM_{2.5}$ estimated using medium and heavy densities of smoke plume.

| | Included | Excluded |
|---|---|---|
| **Clinics** | 161 | 29 |
| **Clinic-days** | 156,575 | 30,165 |
| **Mean patients per clinic** | 100.5 | 74.1 |
| **Missing $PM_{2.5}$ (%)** | 4.46 | 86.41 |
| **Smoke plume days (%)** | 4.03 | 4.84 |
| **EHE days (%)** | 6.91 | 7.11 |
| **Mean $UTCI_{max}$ (°C)** | 30.77 | 30.36 |
| **California / Oregon / Washington** | 113 / 25 / 23 | 19 / 8 / 2 |

**eTable 7. Additive interaction for smoke plume and EHE exposure.**

Four-level joint exposure at lag 0, adjusted for non-wildfire $PM_{2.5}$. Reference: neither exposure. Values in parentheses represent the corresponding 95% confidence intervals.

| Measure | All-cause mortality | All-cause hospitalization |
|---|---|---|
| **RR, smoke plume only** | 1.03 (0.96, 1.11) | 1.01 (0.96, 1.06) |
| **RR, EHE only** | 0.93 (0.88, 0.98) | 1.03 (0.99, 1.06) |
| **RR, both** | 1.80 (1.61, 2.01) | 1.01 (0.93, 1.10) |
| **RERI** | 0.84 (0.63, 1.05) | −0.03 (−0.13, 0.07) |
| **Attributable proportion** | 0.47 (0.40, 0.54) | −0.03 (−0.13, 0.07) |

*Note*. EHE, extreme heat event; RERI, relative excess risk due to interaction; RR, rate ratio.
